# Surface Plasmon Resonance Sensitivity Enhancement Based on Protonated Polyaniline Films Doped by Aluminum Nitrate

**DOI:** 10.3390/bios12121122

**Published:** 2022-12-03

**Authors:** Qais M. Al-Bataineh, Victoria Shpacovitch, Diyar Sadiq, Ahmad Telfah, Roland Hergenröder

**Affiliations:** 1 Leibniz Institut für Analytische Wissenschaften-ISAS-e.V., Bunsen-Kirchhoff-Straße 11, 44139 Dortmund, Germany; 2 Experimental Physics, TU Dortmund University, 44227 Dortmund, Germany; 3 Centre for Material Science and Nanotechnology, Department of Physics, The University of Zakho, Zakho Box. 12, Iraq; 4 Nanotechnology Center, The University of Jordan, Amman 11942, Jordan

**Keywords:** polyaniline (PANI), aluminum nitrate (Al(NO_3_)_3_), complex composite films, surface plasmon resonance (SPR), electrical conductivity

## Abstract

Complex composite films based on polyaniline (PANI) doped hydrochloric acid (HCl) incorporated with aluminum nitrate (Al(NO_3_)_3_) on Au-layer were designed and synthesized as a surface plasmon resonance (SPR) sensing device. The physicochemical properties of (PANI-HCl)/Al(NO_3_)_3_ complex composite films were studied for various Al(NO_3_)_3_ concentrations (0, 2, 4, 8, 16, and 32 wt.%). The refractive index of the (PANI-HCl)/Al(NO_3_)_3_ complex composite films increased continuously as Al(NO_3_)_3_ concentrations increased. The electrical conductivity values increased from 5.10 µS/cm to 10.00 µS/cm as Al(NO_3_)_3_ concentration increased to 32 wt.%. The sensitivity of the SPR sensing device was investigated using a theoretical approach and experimental measurements. The theoretical system of SPR measurement confirmed that increasing Al(NO_3_)_3_ in (PANI-HCl)/Al(NO_3_)_3_ complex composite films enhanced the sensitivity from about 114.5 [Deg/RIU] for Au-layer to 159.0 [Deg/RIU] for Au-((PANI-HCl)/Al(NO_3_)_3_ (32 wt.%)). In addition, the signal-to-noise ratio for Au-layer was 3.95, which increased after coating by (PANI-HCl)/Al(NO_3_)_3_ (32 wt.%) complex composite layer to 8.82. Finally, we conclude that coating Au-layer by (PANI-HCl)/Al(NO_3_)_3_ complex composite films enhances the sensitivity of the SPR sensing device.

## 1. Introduction

Light photons interact with the conduction electrons at the metal/dielectric interface, which generates longitudinal surface waves called surface plasmons [1]. Recently, SPR (surface plasmon resonance)-based sensors have earned growing attention due to their high sensitivity and capability to perform real-time measurements [2,3]. The imaging wide-field SPR microscopy sensor is an optical device that enables the spatiotemporal detection of individual nano-particles in solutions and gas media [4,5,6,7,8,9]. The described wide-field SPR microscopy sensor is based on Kretschmann’s scheme [10] of plasmon excitation. In this scheme, a thin (around 50 nm) planar gold film coats the base of a glass prism and is irradiated by a laser beam. In an attempt to enhance the sensitivity of SPR sensors, various design modifications have been employed, such as the incorporation of different nanostructures [11,12], bimetallic layers [13,14], and conducting and insulating polymer coating [15,16]. Conductive polymer films located on the gold layer have been used to improve the SPR sensitivity [17,18] due to their electrical, optical, and chemical properties [19,20]. F. Usman et al. [21] investigated the sensitivity of SPR-based sensors based on polyaniline/chitosan composite film for detecting low-concentration acetone vapor. They found that polyaniline/chitosan composite film has good selectivity and sensitivity for detecting low-concentration acetone vapor.

Polyaniline (PANI) is an attractive conductive polymer because it exhibits thermal and electrochemical stability, can be relatively easily prepared, and possesses excellent biocompatibility, flexibility, conductivity, and optoelectrical characteristics [17]. PANI composite films are promising candidates for many applications, such as organic solar cells, photodetectors, and gas sensors [22,23,24,25,26]. The high optical response of PANI can be the foremost essential property for optical bio-detectors due to its simplicity, high stability, and cost-effectiveness [27,28,29]. Doping PANI with protonic acids containing different types of counterions enhances electrical conductivity and stability under ambient conditions [30]. Introducing metal ions into the polymer matrix enhances the ionic kinetics of the composite by inducing greater closeness through the metal/polymer blend [31]. In addition, incorporating polymer with aluminum (Al^3+^) ions enhances the electrical conductivity and tuning of the optical properties [32]. Al^3+^ ions could be incorporated with the polymer as intense ions using aluminum nitrate (Al(NO_3_)_3_), aluminum chloride (AlCl_3_), or other ionic forms. However, using aluminum nitrate as incorporation in a polymer matrix is favorable due to the hydrogen bonds between it and the polymer [33]. In addition, aluminum nitrate is an ionic compound composed of aluminum metal and a nitrogen oxoanion that is used for corrosion inhibitors, nitrating agents, and insulating papers [34].

In this research, the SPR microscopy sensor based on (PANI-HCl)/Al(NO_3_)_3_ complex composite layer on Au-layer was designed to enhance the sensitivity of the instrument. (PANI-HCl)/Al(NO_3_)_3_ complex composite films were chosen as a model of SPR application due to their high refractive index, which enhances the SPR signal sensitivity. The sensitivity of the SPR sensor was studied using a theoretical approach and experimental measurements based on imaging a wide-field SPR microscopy sensor. In addition, the structural, chemical, morphological, optical, and electrical properties of (PANI-HCl)/Al(NO_3_)_3_ complex composite films were investigated.

## 2. Materials and Methods

### 2.1. Synthesis Technique

Protonated PANI composite solution was synthesized by dissolving 0.5 g Polyaniline (PANI, emeraldine base, M_W_ = 50,000 g/mol, Sigma Aldrich, Darmstadt, Germany) with 0.07 mL hydrochloric acid (HCl, M_w_ = 36.458 g/mol, Sigma Aldrich) in 100 mL N-Methyl-2-Pyrrolidone (NMP, M_w_ = 99.133 g/mol, Sigma Aldrich) using a magnetic stirrer overnight at 55 °C. The PANI-HCl composite solution was then transferred to a sonication bath for 3 h at 55 °C to reach high homogeneity. The solution was filtered using a centrifuge system and filter paper according to a protocol in the literature [35]. (PANI-HCl)/Al(NO_3_)_3_ complex composite solutions were prepared by adding aluminum nitrate (Al(NO_3_)_3_, 212.996 g/mol, Sigma Aldrich) directly into the PANI-HCl composite solution by the desired amount to achieve the desirable concentrations (0, 2, 4, 8, 16, and 32 wt.%). (PANI-HCl)/Al(NO_3_)_3_ complex composite solutions were homogenized using continuous stirring overnight at room temperature. Next, the complex composite solutions were transferred to a sonication bath for 3 h at 55 °C to reach high homogeneity. (PANI-HCl)/Al(NO_3_)_3_ complex composite films were synthesized by spin coating technique to achieve 250 nm film thickness. The complex composite films were dried at 40 °C in a vacuum oven for 24 h to ensure the total solvent drying and avoid morphology changing. In addition, the (PANI-HCl)/Al(NO_3_)_3_ complex composite film on the gold layer was deposited using the spin coating technique at a higher speed to get a smooth layer with a 30 nm film thickness.

### 2.2. Characterization Techniques

All measurements were performed under ambient conditions. The chemical properties of the complex composite were investigated by studying the vibrational bands obtained from FTIR microscope measurement (HYPERION 3000 Bruker, Karlsruhe, Germany). In addition, the crystal structure was studied using Powder XRD (Malvern Panalytical Ltd., Malvern, UK) using CuKα_1_ ray (λ=0.1540598 nm). Surface wettability was studied using water contact angle measurements for a water droplet (pH = 7) of size 10 µL taken on three occasions. The thermal stability was measured using thermogravimetric analysis (TGA) (NETZSCH Premier Technologies). Optical properties were investigated by analyzing transmittance, and reflectance spectra were measured using a UV–Vis spectrophotometer (Hitachi U-3900H) with a total internal sphere. The electrical conductivity was measured by a 4-point probe (Microworld Inc., New Jersey, USA) connected with a high-resolution multimeter (Keithley 2450 Sourcemeter) for various temperatures from 298–328 K.

### 2.3. Surface Plasmon Resonance (SPR) Measurements

The SPR experimental setup was built up as described previously [8,36]. Glass slides with the same refractive index as a prism (*n* = 1.725) and made from the same type of glass (SF10) were employed as sensing surface carrying slides (glass slides were produced by Hellma Optics, Jena, Germany). Glass slides (sizes 14 × 75 × 1 mm) were coated with 5 nm adhesion layer of Ti and approximately 41–45 nm layer of gold [8]. The deposition was performed using a magnetron-sputtering technique (Innolume, Dortmund, Germany). The gold layer was used as a seed layer on the prism for (PANI-HCl)/Al(NO_3_)_3_ complex composite film. A laser diode (HL6750MG, Thorlabs GmbH, Bergkirchen, Germany; λ = 685 nm) was used to irradiate the (Au-(PANI-HCl)/Al(NO_3_)_3_) layer through a glass prism. The (Au-(PANI-HCl)/Al(NO_3_)_3_) layer was imaged onto a video camera with a CMOS chip (MT9P031 CMOS image sensor chip with a resolution of 5 Mp (megapixel). This chip possesses a pixel size of 2.2 × 2.2 µm) employing macro-objective (Cannon Compact-Macro Lens EF 50 mm 1:2.5). The gold substrate was placed on the glass prism using RI matching immersion liquid (Cargille Laboratories via VWR (*n* = 1.725) to avoid the air gap between the Au layer and prism. The resonance angle represented the minimum reflectivity of the camera image. Polystyrene nanoparticles (PSNPs, refractive index *n* = 1.59 [37]) with an average size of 200 nm were used in SPR measurements.

## 3. Results and Discussion

### 3.1. Physicochemical Properties

**Figure 1:**
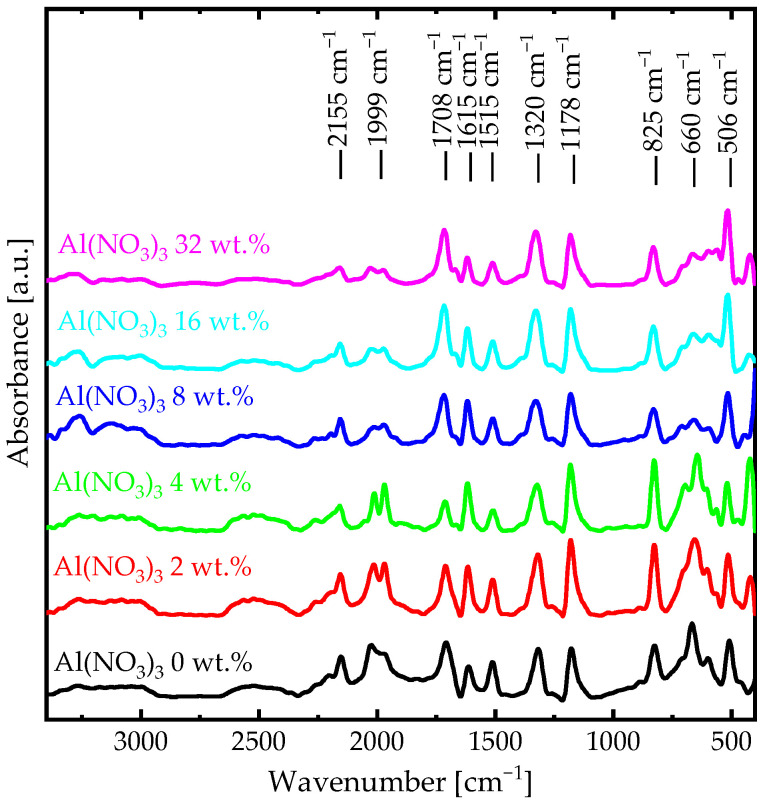
FTIR spectra of (PANI-HCl)/Al(NO_3_)_3_ complex composite films with various Al(NO_3_)_3_ concentrations.

**Figure 2:**
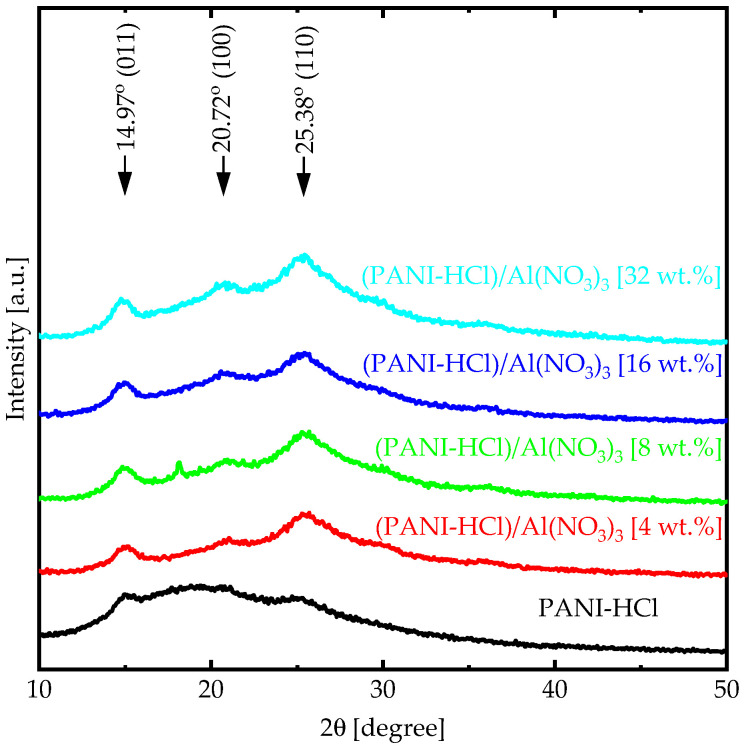
XRD patterns for (PANI-HCl)/Al(NO_3_)_3_ complex composite films for varying Al(NO_3_)_3_ concentrations (wt.%).

The chemical and crystal structure of (PANI-HCl)/Al(NO_3_)_3_ complex composite films was characterized by analyzing the FTIR absorbance spectra (Figure 1, Table 1) and XRD patterns (Figure 2). For PANI-HCl film, the absorption band at 506 cm^−1^ represented the out-of-plane C−H bending vibrations, while the absorption band at 660 cm^−1^ referred to C=N iminoquinone [38,39]. The absorption band at 825 cm^−1^ referred to paradisubstituted aromatic rings indicating polymer formation [30]. Moreover, the absorption band at 1172 cm^−1^ represented the in-plane C−H bending vibrations within the quinoid unit (N=Q=N). The absorption band at 1314 cm^−1^ represented the aromatic C−N stretching vibrations within a secondary aromatic amine group, confirming the protonation of PANI with HCl [35]. In addition, C−N stretching vibrations within benzenoid (N−B−N) and quinoid (N=Q=N) rings appeared at 1515 and 1615 cm^−1^, respectively. The absorption bands between 1999 and 2155 cm^−1^ represented the aromatic C−H stretching vibrations, while the absorption bands after 3000 cm^−1^ represented the N−H stretching vibrations [30,38]. The appearance of an additional peak at 420 cm^−1^ (Al−N) in (PANI-HCl)/Al(NO_3_)_3_ complex composite films confirmed the existence of Al^3+^ ions inside the polymer matrix within Al^3+^ ions connected with the N atoms in the benzoin ring of the PANI or as Al(NO_3_)_3_ [40]. In conclusion, FTIR absorbance spectra confirmed the protonation of PANI by HCl in addition to their interactions with Al(NO_3_)_3_.

The crystalline nature of the PANI is essential because the highly crystalline conductive polymer displays a conductive property [41]. However, PANI crystallinity depends on synthesis conditions in addition to acid dopants [42]. The XRD patterns for PANI-HCl film showed broad diffraction peaks at 2θ of 14.97°, 20.72°, and 25.38° associated with the crystallographic plane (200), (100), and (110), respectively, which indicated the low degree of crystallinity (Figure 2). All peaks were in good agreement with the literature [43]. Moreover, the broad peak between 10° and 35° and the three diffraction peaks confirmed that PANI-HCl film had a semi-crystalline nature. According to Bhadra and Khastgir [44], PANI has semi-crystalline nature with two phases; the crystal phase, in which the polymer chains are ordered in the close-packed array, and the amorphous phase, where the polymer chains do not order. Introducing Al(NO_3_)_3_ into PANI-HCl film led to an increase in the (200) and (110) plane intensities, which means that the Al^3+^ interacted with the PANI-HCl matrix at N atoms and consequently changed the crystal structure of the complex composite films by making it ordered in the close-packed array.

**Figure 3:**
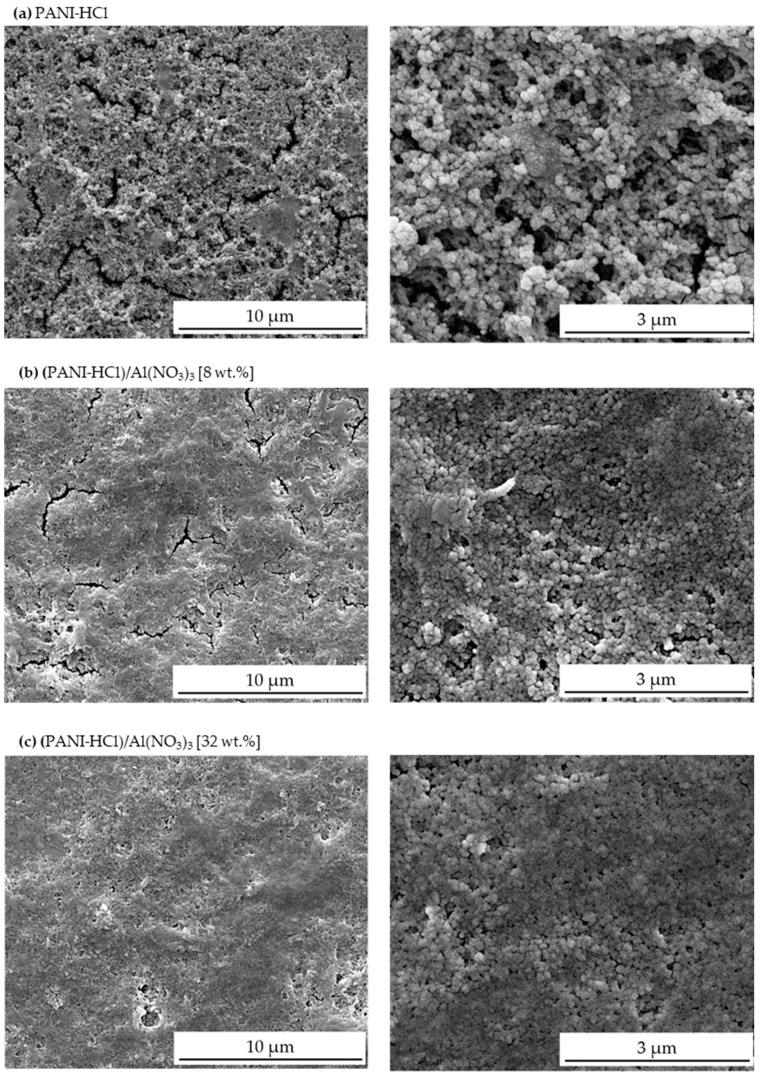
SEM micrographs from (PANI-HCl)/Al(NO_3_)_3_ complex composite films with Al(NO_3_)_3_ concentration (0, 8, and 32 wt.%).

Figure 3 shows the SEM images for (PANI-HCl)/Al(NO_3_)_3_ complex composite films at Al(NO_3_)_3_ concentrations of 0, 8, and 32 wt.%. The PANI-HCl film showed a rod-like shape with an average diameter of 130 nm (Figure 3a). Adding Al(NO_3_)_3_ into the PANI-HCl films by 8% and 32% led to a decrease in the average diameter of rod-like shapes to 92 nm and 45 nm, respectively (Figure 3b,c). In addition, adding Al(NO_3_)_3_ to the PANI-HCl films also increased the film surface’s smoothness.

**Figure 4:**
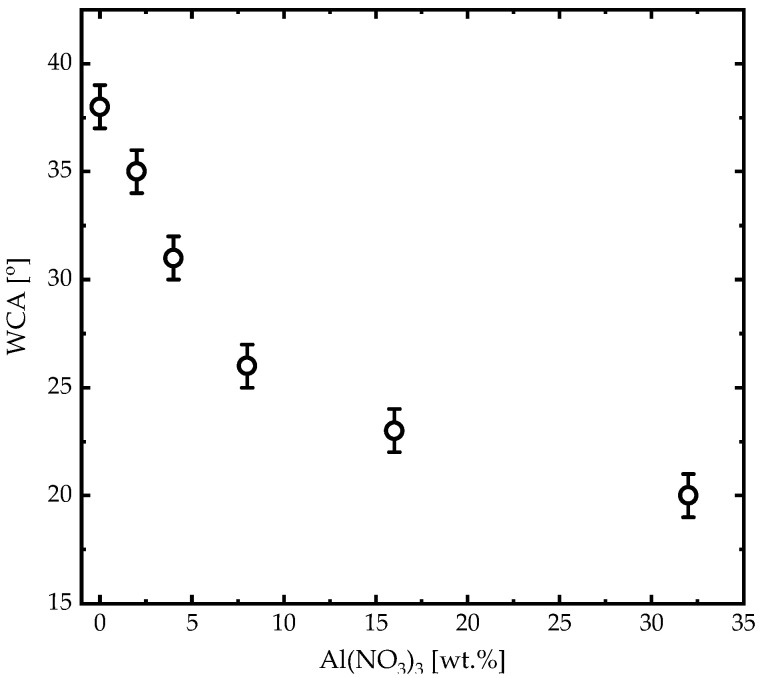
Water contact angles of (PANI-HCl)/Al(NO_3_)_3_ complex composite films as a function of Al(NO_3_)_3_ concentration.

The surface wettability, in addition to SEM micrographs of the (PANI-HCl)/Al(NO_3_)_3_ complex composite films with various Al(NO_3_)_3_ concentrations, was studied to understand the physicochemical interactions between polymer matrix and Al(NO_3_)_3_ (Figure 4). The WCA for PANI-HCl film was 38°, which means that the PANI-HCl film exhibited a hydrophilic nature [45]. Increasing Al(NO_3_)_3_ in (PANI-HCl)/Al(NO_3_)_3_ complex composite films reduced the WCA continuously until it reached 20° at Al(NO_3_)_3_ concentration of 32 wt.%, which means that increasing Al(NO_3_)_3_ concentration in (PANI-HCl)/Al(NO_3_)_3_ complex composite films enhanced the hydrophilicity nature of the film surface, which was attributed to a decrease in the average diameter of rod-like shapes and a reduction in the surface roughness.

**Figure 5:**
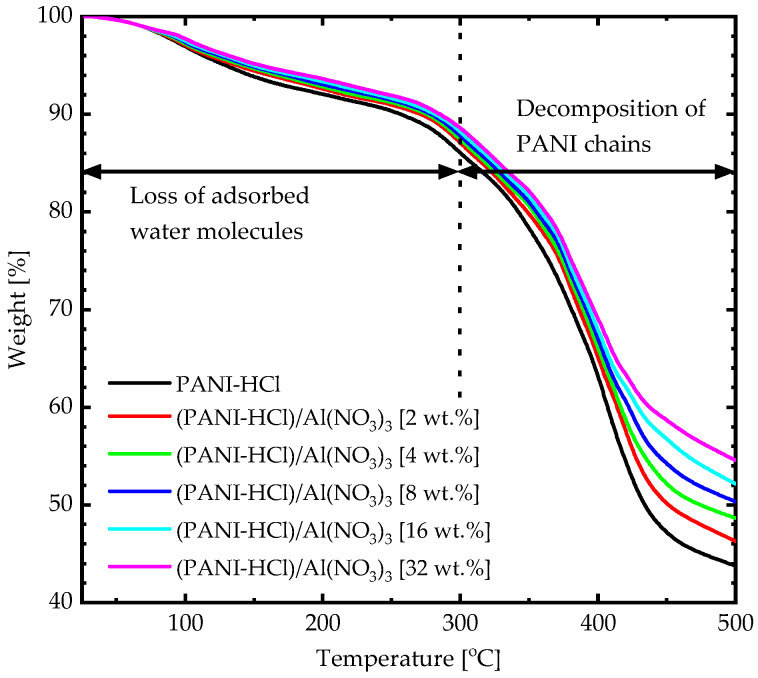
TGA curves of (PANI-HCl)/Al(NO_3_)_3_ complex composite films with various Al(NO_3_)_3_ concentrations.

The thermal stability of (PANI-HCl)/Al(NO_3_)_3_ complex composite films was investigated using Thermogravimetric Analysis (TGA) at temperatures up to 500 °C (Figure 5). TGA curves of all composite films underwent two stages of weight loss. In the first stage, the PANI-HCl film lost around 13% of its weight when the temperature rose from 75 °C to 300°C, which was attributed to solvent evaporation. In addition, the second stage showed a considerable weight loss (around 42%) as the temperature rose from 300 °C to 500 °C, which was attributed to the PANI chains decomposition [30]. The weight loss of all composite films shifted toward higher temperatures as increasing Al(NO_3_)_3_ concentrations increased, which was attributed to an increase in the strength of physicochemical bonding density in the (PANI-HCl)/Al(NO_3_)_3_ complex composite films [46]. To conclude, (PANI-HCl)/Al(NO_3_)_3_ complex composite films were thermally stable within the temperatures at the optical, electrical, and SPR applications.

### 3.2. Optical Characterizations

**Figure 6:**
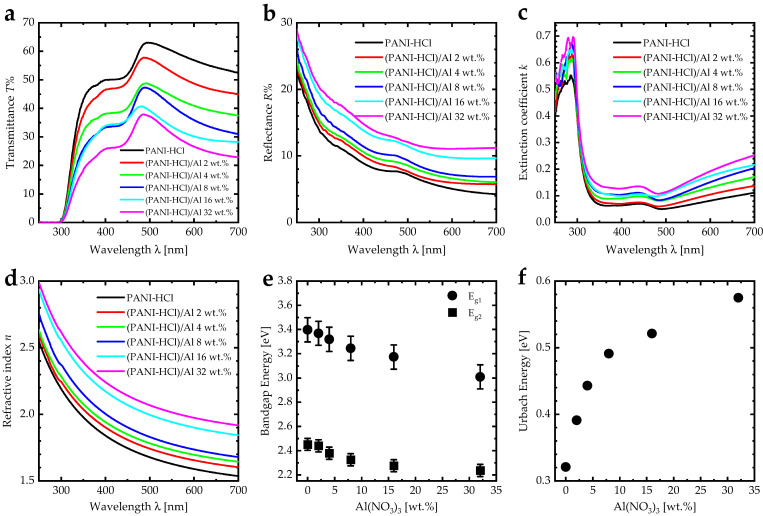
(**a**) Transmittance spectra, (**b**) reflectance spectra, (**c**) extinction coefficient spectra, (**d**) refractive index spectra, (**e**) bandgap energy, and (**f**) Urbach energy for (PANI-HCl)/Al(NO_3_)_3_ complex composite films with varying Al(NO_3_)_3_ concentration.

UV-Vis transmittance, reflectance spectra, and the corresponding optical properties of (PANI-HCl)/Al(NO_3_)_3_ complex composite films are illustrated in Figure 6. The transmittance spectra of the PANI-HCl film exhibited the first steep escalating values from around 0.0% up to 47.5% as the wavelength increased from 300 nm to 370 nm, and the second steep escalating values from 51.0% up to 63.0% as the wavelength increased from 440 nm to 490 nm. In addition, by increasing the wavelength from 490 nm to 700 nm, the transmittance decreased from 63.0% to 52.5% (Figure 6a). Introducing Al(NO_3_)_3_ into the PANI-HCl films reduced the transmittance values in the visible region with non-linear behavior. For instance, the transmittance value of PANI-HCl film at 550 nm was 60%, which decreased to 31% for (PANI-HCl)/Al(NO_3_)_3_ complex composite film with 32 wt.% of Al(NO_3_)_3_. Additionally, the sharp decrease in the transmittance of the PANI-HCl film in the UV region (300–370 nm) indicated strong electronic transitions within a high-absorption region. The inclusion of Al(NO_3_)_3_ into the PANI-HCl films led to a red shift in the absorption edge, which means increased electron transitions between the PANI and Al^+3^ ions. The reflectance spectra of (PANI-HCl)/Al(NO_3_)_3_ complex composite films with different Al(NO_3_)_3_ concentrations are presented in Figure 6b. The reflectance continuously decreased as the wavelength increased from 250 nm to 700 nm. Incorporation of Al(NO_3_)_3_ into the PANI-HCl films increased reflectance. As expected, the reflectance spectrum showed the opposite trend to the transmittance spectrum.

**Figure 7:**
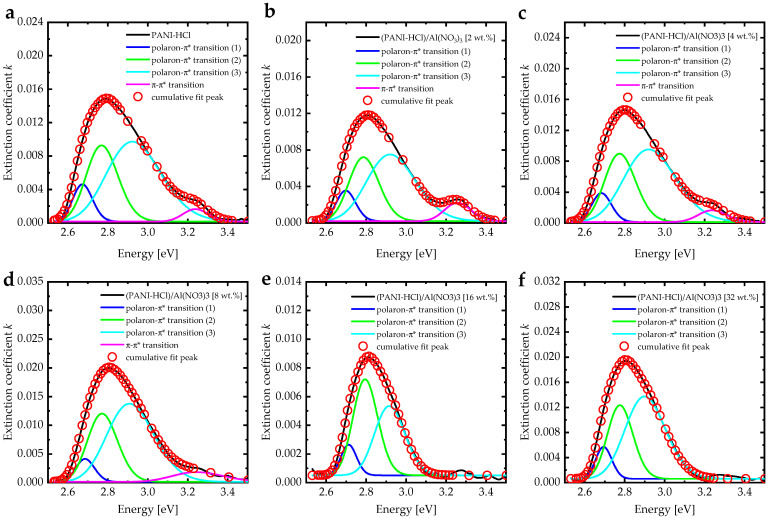
The extinction coefficient band de-convoluted by fitting the spectral envelope to four Gaussian peaks of (PANI-HCl)/Al(NO_3_)_3_ complex composite films with varying Al(NO_3_)_3_ concentrations: (**a**) 0, (**b**) 2, (**c**) 4, (**d**) 8, (**e**) 16, and (**f**) 32 wt.%.

The extinction coefficient (*k*) can be calculated using *k* = αλ/4π, where α is the absorption coefficient, given by α = (1/*d*)ln((1 − R)/T), where R is the reflectance, T is the transmittance, and d is the film thickness [47]. The extinction coefficient spectra had a noticeable drop in the wavelength range of 250–350 nm, indicating strong electronic transitions within a high-absorption region (Figure 6c). However, the increase in Al(NO_3_)_3_ concentration in (PANI-HCl)/Al(NO_3_)_3_ complex composite films increased the extinction coefficient values in the visible region, indicating that more photon energy was lost by absorption and scattering. Additionally, the extinction coefficient spectra of (PANI-HCl)/Al(NO_3_)_3_ complex composite films exhibited overlapping bands in the region of 360–500 nm, representing the π-π* transition within the benzoin ring and localized polarons (polaron-π*) transition [48]. The increase in the extinction coefficient values after 500 nm was attributed to the π-polaron transition, which occurred between 820–840 nm [49]. The extinction coefficient peak was de-convoluted by fitting the spectral envelope to four Gaussian peaks as a function of energy (Figure 7) [50]. The fitting was converged with an R^2^ of about 0.99. De-convoluted was tested by fitting the extinction coefficient band to Lorentzian, bi-Gaussian, and Voigt functions, but the fitting converged more poorly than the Gaussian fitting. Before fitting, the baseline correction was performed according to the algorithm described in [51]. For the PANI-HCl film, the first peak at 3.24 eV represented the π-π* transition. In addition, the other three peaks at 2.92 eV, 2.77 eV, and 2.67 eV represented the polaron-π* transition [48]. The broad distribution of the polaron-π* transition can be attributed to how the rod-like shape increased the interaction between polarons and consequently improved the interband transitions [52]. Adding Al(NO_3_)_3_ into the PANI-HCl led to a decrease in the π-π* transition band until it disappeared at 16 wt.% of Al(NO_3_)_3_, which can be attributed to the interaction between the nitrogen atom in the benzoin ring of PANI and the Al^3+^ ions (Figure 1).

The refractive index values (*n*) can be calculated using $n=\left(1+R)/(1-R\right)+\sqrt{\left(4R/{\left(1-R\right)}^{2}\right)-k^{2}}$ and fitted to the Cauchy model, which is given by *n* = *A+B/λ^2^*, where *A* and *B* are constants [53]. The refractive index for the PANI-HCl film decreased from 2.55 to 1.54 as the incident photon wavelength increased from 250 nm to 700 nm (Figure 6d). Moreover, increasing the Al(NO_3_)_3_ concentration in (PANI-HCl)/Al(NO_3_)_3_ complex composite films increased the refractive index due to the interaction between Al^3+^ ions and the PANI interaction that may increase the compositional density of the composite films [54].

Tauc plots were performed to investigate the bandgap energy of (PANI-HCl)/Al(NO_3_)_3_ complex composite films by plotting (α*h*ν)^2^ versus *h*ν, according to the equation (α*h*ν)^2^ = *β(hν − E_g_)* for direct band-gap semiconductors. All the films exhibited two bandgap energies; the first band-gap energy (*E_g1_*) decreased from 3.40 eV to 3.01 eV, representing the π-π* electron transition within the benzenoid (B) ring, while the second bandgap energy (*E_g2_*) decreased from 2.45 eV to 2.24 eV, representing localized polarons (polaron-π*) (quinoid, Q) electron transition (Figure 6e). The decrease in the bandgap energies as increasing Al(NO_3_)_3_ can be attributed to generating new energy levels between the HUMO and LOMO because of the disorder in the structure of the composite films [55]. Urbach energy, *E_U_* is considered an essential method for studying the degree of disorder in films, which is calculated using α = α_0_ exp(*hν*/*E_U_*), where: α_0_ is a constant [56,57]. The Urbach energy for the PANI-HCl film was 321 meV, which was consistent with the Urbach energy for PANI-CSA in the literature [58]. An increase of the concentration of Al(NO_3_)_3_ in the PANI-HCl matrix to 32 wt.% led to the growth of Urbach energy to 575 meV, suggesting the highest disorder at this concentration.

### 3.3. Electrical Conductivity

**Figure 8:**
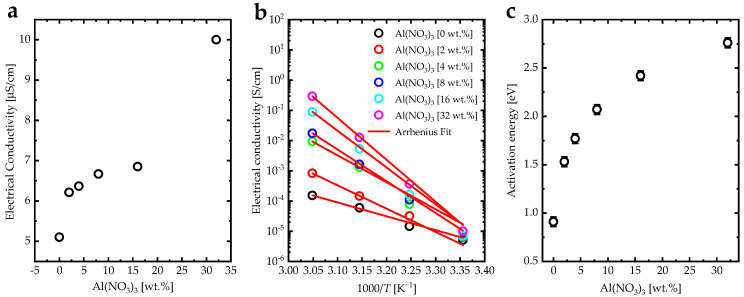
(**a**) Electrical conductivity vs. Al(NO_3_)_3_ concentration [wt.%], (**b**) electrical conductivity vs. 1000/T [K^−1^], and (**c**) activation energy vs. Al(NO_3_)_3_ concentration [wt.%] for (PANI-HCl)/Al(NO_3_)_3_ complex composite films.

Figure 8a illustrates the electrical conductivity (σ) variations of the (PANI-HCl)/Al(NO_3_)_3_ complex composite films as a function of Al(NO_3_)_3_ concentration. The electrical conductivity of the PANI-HCl film was 5.10 µS/cm, which can be attributed to the polaron states in PANI and doping by HCl, which has an essential influence on polymer oxidation. However, the conductivity of the (PANI-HCl)/Al(NO_3_)_3_ complex composite films can be attributed to the superposition of polaron states in PANI and the effects of the Al^3+^ ions [59]. An increase of Al(NO_3_)_3_ concentration in the PANI-HCl films to 32% led to the growth of conductivity up to 10.00 µS/cm due to the increase of Al^3+^ ion density, which was determined through the conduction process formed by the incorporation Al(NO_3_)_3_ with PANI by ionic interactions [60]. The electrical conductivity of (PANI-HCl)/Al(NO_3_)_3_ complex composite films increased as the temperature increased (Figure 8b). This can be attributed to the thermal activation of ion mobility and electron hopping [61]. In addition, the electrical conductivity values as a function of 1000/T(K) for (PANI-HCl)/Al(NO_3_)_3_ complex composite films were fitted to the Arrhenius function (σ = σ_0_ exp(−*E_a_*/*K_B_T*)) [32]. The activation energy (*E_a_*) of (PANI-HCl)/Al(NO_3_)_3_ complex composite films increased with increasing Al(NO_3_)_3_ concentration (Figure 8c).

**Figure 9:**
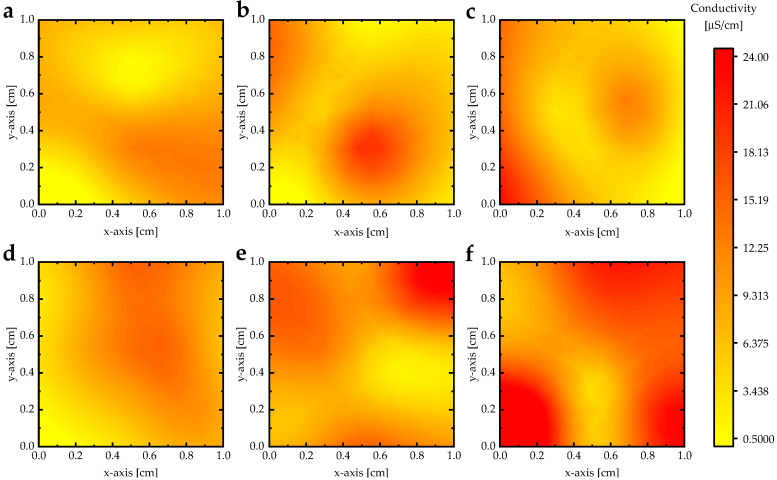
The conductivity maps (1 cm × 1 cm) of (PANI-HCl)/Al(NO_3_)_3_ complex composite films with Al(NO_3_)_3_ concentrations: (**a**) 0 wt.%, (**b**) 2 wt.%, (**c**) 4 wt.%, (**d**) 8 wt.%, (**e**) 16 wt.%, and (**f**) 32 wt.%.

The conductivity maps (1 cm × 1 cm) of (PANI-HCl)/Al(NO_3_)_3_ complex composite films are shown in Figure 9. The conductivity of the PANI-HCl film showed a slight alteration of electrical conductivity across the film (Figure 9a), which can be attributed to the surface morphology and growth process quality. Including Al(NO_3_)_3_ in the PANI-HCl film increased the electrical conductivity across the film. Moreover, the electrical conductivity of (PANI-HCl)/Al(NO_3_)_3_ complex composite films had a semi-homogeneous distribution (Figure 9b–f).

### 3.4. Surface Plasmon Resonance (SPR) Measurements

**Figure 10:**
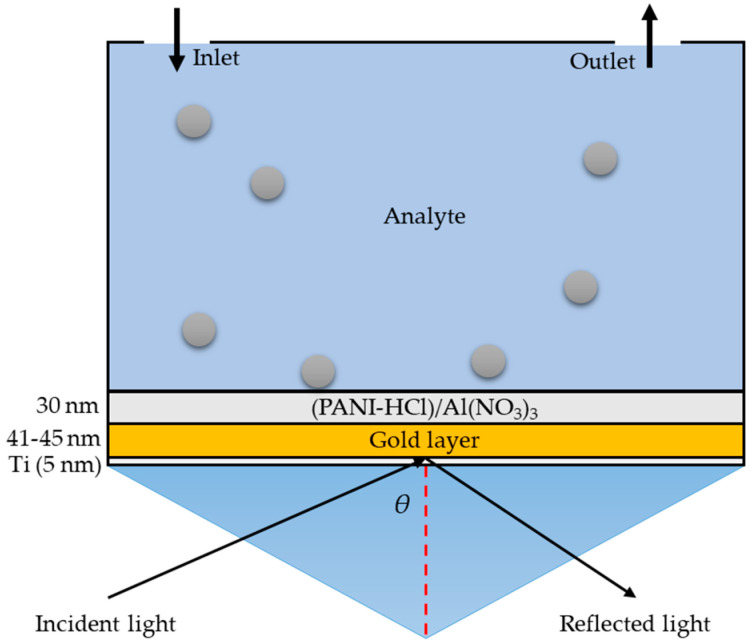
Kretschmann configuration employed in SPR-microscopy sensor: a glass prism, titanium adhesion layer (5 nm), gold layer (41–45 nm), and (PANI-HCl)/Al(NO_3_)_3_ complex composite layer (30 nm) with an analyte for sensing biomolecules.

This section describes a model built using real SPR microscopy sensor measurements. The (PANI-HCl)/Al(NO_3_)_3_ complex composite films were used to improve the SPR sensitivity [62]. The model was composed of a glass prism, a titanium adhesion layer (5 nm), a gold layer (41–45 nm), and a (PANI-HCl)/Al(NO_3_)_3_ complex composite layer (30 nm) (Figure 10). Additionally, (PANI-HCl)/Al(NO_3_)_3_ complex composite films were connected with the analyte (water) for detecting polystyrene nanoparticles. The n of each complex composite layer was determined experimentally (Figure 6) at a wavelength of 685 nm in order to match the wavelength in the theoretical model to that used in the experimental SPR measurements (Table 1) since W. Mukhtar et al. [63] concluded that visible light in the range of 600–700 nm has a high sensitivity for SPR measurements.

**Figure 11:**
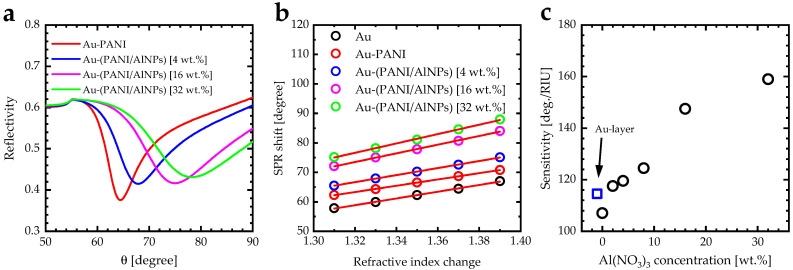
(**a**) SPR reflectivity curve for Kretschmann configuration of the prism-gold-(PANI-HCl)/Al(NO_3_)_3_ systems for selected Al(NO_3_)_3_ content [wt.%], (**b**) SPR angle shift versus the refractive index change of selected Al(NO_3_)_3_ content [wt.%], and (**c**) Sensitivity variation as a function of Al(NO_3_)_3_ concentration [wt.%] of prism-gold-(PANI-HCl)/Al(NO_3_)_3_ system.

Figure 11a shows the theoretical reflectivity values of the Au layers for selected concentrations of Al(NO_3_)_3_ as a function of beam angle using WINSPALL software [64]. The inclusion of Al(NO_3_)_3_ in (PANI-HCl)/Al(NO_3_)_3_ complex composite films increased the incident photon angle at minimum reflectivity (Table 2). The sensitivity of the SPR sensor based on Au-((PANI-HCl)/Al(NO_3_)_3_) layers was calculated by *S = Δθ/Δn*, where: *Δθ* represented the SPR angle shift [degree] and *Δn* was refractive index change [refractive index unit: RIU]. The linear fit of the SPR angle shift as a function of refractive index changes represented the sensitivity of the SPR sensor (Figure 11b). Inclusion of Al(NO_3_)_3_ in the PANI-HCL films enhanced the SPR sensitivity from 114.5 [Deg/RIU] for pare Au-layer to 159.0 [Deg/RIU] for Au-((PANI-HCl)/Al(NO_3_)_3_) layers for 32 wt.% of Al(NO_3_)_3_ (Figure 11c). In addition, another measurement scale for getting a clear concept of the sensing efficiency is the figure of merit (FOM) of the sensor. This is determined as the ratio of the sensitivity to the sensor’s linewidth (LW), which is given by FOM = S/LW [65]. According to the definition of FOM, increasing the sensitivity led to enhancing the FOM. However, the higher increases in the LW decreased the FOM values. Therefore, the FOM decreased as Al(NO_3_)_3_ concentrations decreased in the PANI-HCl.

**Figure 12:**
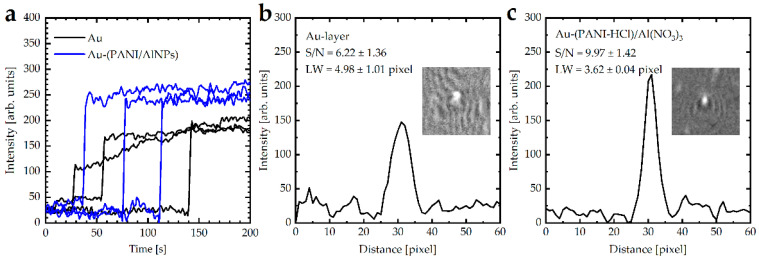
(**a**) Time dependence of the intensity in the middle of the three bright spots for PSNPs by Au-layer and Au-((PANI-HCl)/Al(NO_3_)_3_) layers with Al(NO_3_)_3_ concentration of 36 wt.%., (**b**) line profile plot of PSNPs detected by Au-layer, and (**c**) line profile plot of PSNPs detected by Au-((PANI-HCl)/Al(NO_3_)_3_) layers. The inset in (**b**,**c**) represents the typical processed image of bound polystyrene particles to the surface, which showed bright spots surrounded by a relatively low-intensity background. Three replicates were performed.

The detection of spherical PSNPs (200 nm) by Au layer and Au-((PANI-HCl)/Al(NO_3_)_3_) layers with Al(NO_3_)_3_ concentration of 32 wt.% were studied experimentally. After recording images using a CMOS camera, ImageJ software [67] was applied for image processing, including averaging and background subtraction. Figure 12a illustrates the relative intensity before the binding of PSNPs with the surface and the sudden increase of the local intensity level in a moment of a nano-particle binding. The signal intensity to the noise intensity of the Au-((PANI-HCl)/Al(NO_3_)_3_) layers was higher than the signal intensity to the noise intensity of the Au layer. The performance of the SPR measurement was investigated by calculating the signal-to-noise ratio (S/N) in addition to the linewidth in the line profile plot in the x-direction parallel to the surface plasmon propagation vector for three individual bound PSNPs to the surface presented in the inset [56] (Figure 12b,c). The average signal-to-noise ratio for Au-layer was 6.22, which increased after coating with (PANI-HCl)/Al(NO_3_)_3_ complex composite layer to 9.97. This means (PANI-HCl)/Al(NO_3_)_3_ that complex composite films enhance the sensitivity of SPR measurement for particle detection. In addition, it was clearly seen that the average linewidth (LW) of the recorded image decreased from 4.98 pixels to 3.62 pixels by using the Au-((PANI-HCl)/Al(NO_3_)_3_) layers, which means that not only were the sensitivity and SNR improved, but the spatial resolution of the recorded image was also enhanced.

The sensor can also be used as a classical—not as a nano-particle imaging—SPR instrument. In this case, a critical parameter in classical SPR is the limit of detection (LOD), which is defined as the smallest concentration giving the output signal and given by LOD = 3σ/S, where: σ is the noise, and S is the sensitivity of the sensor [68]. The LOD value for the bare Au layer was 0.12 RIU, which decreased to 0.08 RIU after coating with (PANI-HCl)/Al(NO_3_)_3_ with a concentration of 32 wt.%. This means that coating the Au layer with (PANI-HCl)/Al(NO_3_)_3_ can detect fewer concentrations than the bare Au layer.

## 4. Conclusions

Synthesized (PANI-HCl)/Al(NO_3_)_3_ complex composite films were designed as a special coating for a wide-field SPR microscopy sensor chip. The structural, morphological, optical, and electrical properties of (PANI-HCl)/Al(NO_3_)_3_ complex composite films in various Al(NO_3_)_3_ concentrations were investigated and analyzed. FTIR spectra of (PANI-HCl)/Al(NO_3_)_3_ complex composite films confirmed the existence of Al^3+^ ions inside the polymer matrix within Al^3+^ ions connected with the N atoms in the benzoin ring of the PANI or existing as Al(NO_3_)_3_. In addition, the inclusion of Al(NO_3_)_3_ into the PANI-HCl film increased the (011) and (110) plane intensities, indicating that Al^3+^ interacted with the PANI-HCl matrix at N atoms and consequently changed the crystal structure of the complex composite films. SEM images showed that the PANI-HCl film had a rod-like shape with an average diameter of 130 nm. Adding Al(NO_3_)_3_ into the PANI-HCl films by 8% and 32% decreased the average diameter of the rod-like shapes to 92 nm and 45 nm, respectively. Moreover, an increase of Al(NO_3_)_3_ concentration in (PANI-HCl)/Al(NO_3_)_3_ complex composite films enhanced the hydrophilicity of the film surface. Growing Al(NO_3_)_3_ concentration in (PANI-HCl)/Al(NO_3_)_3_ complex composite films leads to the rise of the refractive index of the complex composite film. Increasing the Al(NO_3_)_3_ concentration in the PANI-HCl films to 32% elevated the conductivity up to 10.00 µS/cm, which can be attributed to the growing Al^3+^ ion density. The sensitivity of the SPR wide-field microscopy sensor was investigated using a theoretical approach and experimental measurements. The modeling (simulation) of SPR measurements confirmed that the inclusion of growing concentrations of Al(NO_3_)_3_ in (PANI-HCl)/Al(NO_3_)_3_ complex composite films enhanced sensor sensitivity from around 114.5 [Deg/RIU] for Au-layer to 159.0 [Deg/RIU] for Au-((PANI-HCl)/Al(NO_3_)_3_ (32 wt.%)). In addition, the signal-to-noise ratio for Au-layer was 6.22, which reached 9.97 after coating by (PANI-HCl)/Al(NO_3_)_3_ (32 wt.%) complex composite layer. In addition, the FOM of the bare Au layer was 79.51 RIU^−1^, which decreased as Al(NO_3_)_3_ concentrations increased in the PANI-HCl to 7.23 RIU^−1^. The LOD value for the bare Au layer was 0.12 RIU, which decreased to 0.08 RIU after coating with (PANI-HCl)/Al(NO_3_)_3_ with a concentration of 32 wt.%. This means that coating the Au layer with (PANI-HCl)/Al(NO_3_)_3_ can detect fewer concentrations than the bare Au layer.

## Figures and Tables

**Table 1:** Vibrational bands of (PANI-HCl)/Al(NO_3_)_3_ complex composite film for different Al(NO_3_)_3_ concentrations.

Absorption Bands	Al(NO_3_)_3_ 0 wt.%	Al(NO_3_)_3_ 2 wt.%	Al(NO_3_)_3_ 4 wt.%	Al(NO_3_)_3_ 8 wt.%	Al(NO_3_)_3_ 16 wt.%	Al(NO_3_)_3_ 32 wt.%
out-of-plane C−H bending	506	510	515	517	519	521
C=N iminoquinone	660	658	656	652	650	646
paradisubstituted aromatic rings	825	827	828	829	832	835
in-plane C−H bending vibrations	1178	1178	1178	1184	1184	1184
aromatic C−N stretching	1320	1320	1320	1329	1334	1334
C−N stretching (N−B−N)	1515	1514	1511	1511	1511	1511
C−N stretching (N=Q=N)	1615	1615	1615	1615	1615	1615

**Table 2:** Optical constant values, SPR angle, and SPR sensitivity of the gold film, and (PANI-HCl)/Al(NO_3_)_3_ complex composite film for different Al(NO_3_)_3_ concentrations at a wavelength of 685 nm.

Al(NO_3_)_3_ Concentration [wt.]	Refractive Index *n*	Extinction Coefficient *k*	SPR Angle [°]	SPR Sensitivity [deg/RIU]	Figure of Merit (FOM) [RIU^−1^]
Gold layer	0.15 [66]	4.91 [66]	59.9	114.5	79.51
0	1.51	0.11	64.3	107.0	8.99
2	1.61	0.13	67.0	117.5	8.64
4	1.63	0.17	67.9	119.5	6.91
8	1.67	0.19	69.1	124.5	6.66
16	1.87	0.21	75.0	147.5	7.45
32	1.96	0.24	78.2	159.0	7.23

## Data Availability

Not applicable.
